# Performance of resistive index and semi-quantitative power doppler ultrasound score in predicting acute kidney injury: A meta-analysis of prospective studies

**DOI:** 10.1371/journal.pone.0270623

**Published:** 2022-06-28

**Authors:** Qiong Wei, Yu Zhu, Weifeng Zhen, Xiaoning Zhang, Zhenhua Shi, Ling Zhang, Jiuju Zhou

**Affiliations:** Department of Intensive Care Unit, PLA 983rd Hospital, Tianjin, China; Stanford University School of Medicine, UNITED STATES

## Abstract

This study aimed to assess the predictive value of the renal resistive index (RRI) and power Doppler ultrasound (PDU) on subsequent acute kidney injury (AKI) risk using a meta-analytic approach. We searched eligible studies in PubMed, EmBase, and the Cochrane library from inception until August 2021. The parameters included the sensitivity, specificity, positive and negative likelihood ratios (PLR and NLR), diagnostic odds ratio (DOR), and area under the receiver operating characteristic curves (AUC). Twenty-three prospective studies involving 2,400 patients were selected. The pooled sensitivity and specificity of the RRI and PDU were 0.76 and 0.79, and 0.64 and 0.90, respectively. The pooled PLR and NLR were 3.64 and 0.31, and 6.58 and 0.40 for the RRI and PDU, respectively. The DORs of the RRI and PDU for predicting AKI were 11.76, and 16.32, respectively. The AUCs of the RRI and PDU for predicting AKI were 0.83, and 0.86, respectively. There were no significant differences between the RRI and PDU for predicting AKI in terms of sensitivity, PLR, NLR, DOR, and AUC. The specificity of the RRI was lower than that of the PDU for predicting AKI. This study found that the predictive performance of the RRI and PDU from the Doppler ultrasound for AKI was similar, which need to be further verified based on the direct comparison results.

## Introduction

Acute kidney injury (AKI) is defined as an abrupt decrease and increase in the glomerular filtration rate and serum creatinine, respectively, which are attributed to acute kidney diseases in the process of renal disease [1, 2]. Nowadays, AKI is a common condition for critically ill patients in nearly all fields of medicine [3, 4]. A meta-analysis of 312 studies found the prevalence of AKI and the related mortality rate were 21.6%, and 23.9% in adults worldwide [5]. Abnormal renal blood perfusion plays an important role in the progression of AKI with complicated pathophysiological properties [6]. The diagnosis of AKI involves determining whether outflow tract obstruction, hydronephrosis or a distended bladder are present, which could be revealed by renal ultrasound. Therefore, early diagnosis and intervention in AKI is important, and alternative therapies should be administered to improve the prognosis of patients with AKI.

Ultrasound has already been utilized to evaluate renal perfusion, and the Renal Resistive Index (RRI) has been regarded as an important index to assess renal perfusion [7, 8]. The RRI is calculated from the ratio of the velocities of arterial perfusion throughout the cardiac phase, which is strictly associated with persistent AKI [9]. Meanwhile, the Power Doppler Ultrasound (PDU) determines the velocity of red blood cells and its power on the basis of the amount of blood present. Moreover, a study found that the semi-quantitative PDU scores could predict delayed graft function after renal transplantation, which could assess the severity and prognosis of AKI [10]. However, no studies have compared the predictive performance of the RRI and PDU for AKI in patients with critical illnesses. Therefore, we performed this study to compare the predictive performance of RRI and semi-quantitative PDU score in assessing AKI in patients with various comorbidities.

## Materials and methods

### Data sources, search strategy, and selection criteria

The protocol of this systematic review and meta-analysis was registered at INPLASY (ID: INPLASY202220101). The Preferred Reporting Items for Systematic Reviews and Meta-Analysis Statement published in 2020 was used as a guide for the analysis and reporting methods in this study (S1 Checklist) [11]. The databases of PubMed, EmBase, and the Cochrane library were systematically searched for studies from inception through August 2021, and the following search terms were applied: "acute kidney injury" AND ("Doppler" OR "ultrasound"). We also reviewed related review articles and original research articles to identify additional included studies.

Two reviewers independently performed the literature search and study selection following a standard flow, and disagreement was resolved through discussion until the reviewers reached a consensus. Studies were included if the following inclusion criteria were met: (1) Patients: all of included patients undergoing critically illness or postoperative conditions; (2) Predictive tools: RRI or PDU; (3) Outcome: true positive, false positive, false negative, true negative, or data that could be used to calculate these values; and (4) Study design: the study had to have a prospective study design.

### Data collection and quality assessment

Data extraction and quality assessment were independently performed by 2 reviewers, and inconsistencies between the reviewers were resolved by an additional reviewer who reviewed the original article independently. The following information were extracted: first author’s name, publication year, country, setting, sample size, age of patients, proportion of males, disease status, index and cutoff values, number and proportion of patients with AKI, true positive, false positive, false negative, and true negative. The Quality Assessment of Diagnostic Accuracy Studies 2 was applied to assess the quality of the selected studies using the following items: risk of bias (patient selection, index test, reference standard, flow and timing) and applicability concerns (patient selection, index test, reference standard) [12].

### Statistical analysis

The number of true positives, false negatives, false positives, and true negatives was presented in each original study. The diagnostic odds ratio (DOR) and the area under the receiver operating characteristic curve (AUC) were then assessed to find the overall accuracy. The sensitivity, specificity, positive likelihood ratio (PLR), and negative likelihood ratio (NLR) were also assessed. The pooled parameters were calculated using the bivariate generalized linear mixed model and random-effects model [13–15]. The heterogeneity across the included studies were assessed using the *I*^*2*^ and Q statistic, and significant heterogeneity was considered as *I*^*2*^ > 50.0% or *P* < 0.10 [16, 17]. The indirect comparison of predictive performance between the RRI and PDU were illustrated, and the ratio with 95% confidence interval (CI) between the RRI and PDU was calculated [18]. If a 95% CI for the ratio included the value of 1, there was insufficient evidence to conclude that the predictive performance of the RRI and PDU were significantly different. Subgroup analyses for the predictive parameters were performed based on the country, mean age of patients, proportion of males, and disease status, and the differences between the RRI and PDU or between subgroups were also determined [18]. The publication bias was assessed using funnel plots and Deeks’ asymmetry tests [19, 20], and the trim and fill method was used to calculate the adjusted DOR if significant publication bias was observed [21]. All *P* values for the pooled results were two-sided, and the significance level was set at 0.05. All analyses in our study were performed using software STATA (version 10.0; Stata Corporation, College Station, TX, USA).

## Results

### Literature search

**Fig 1:**
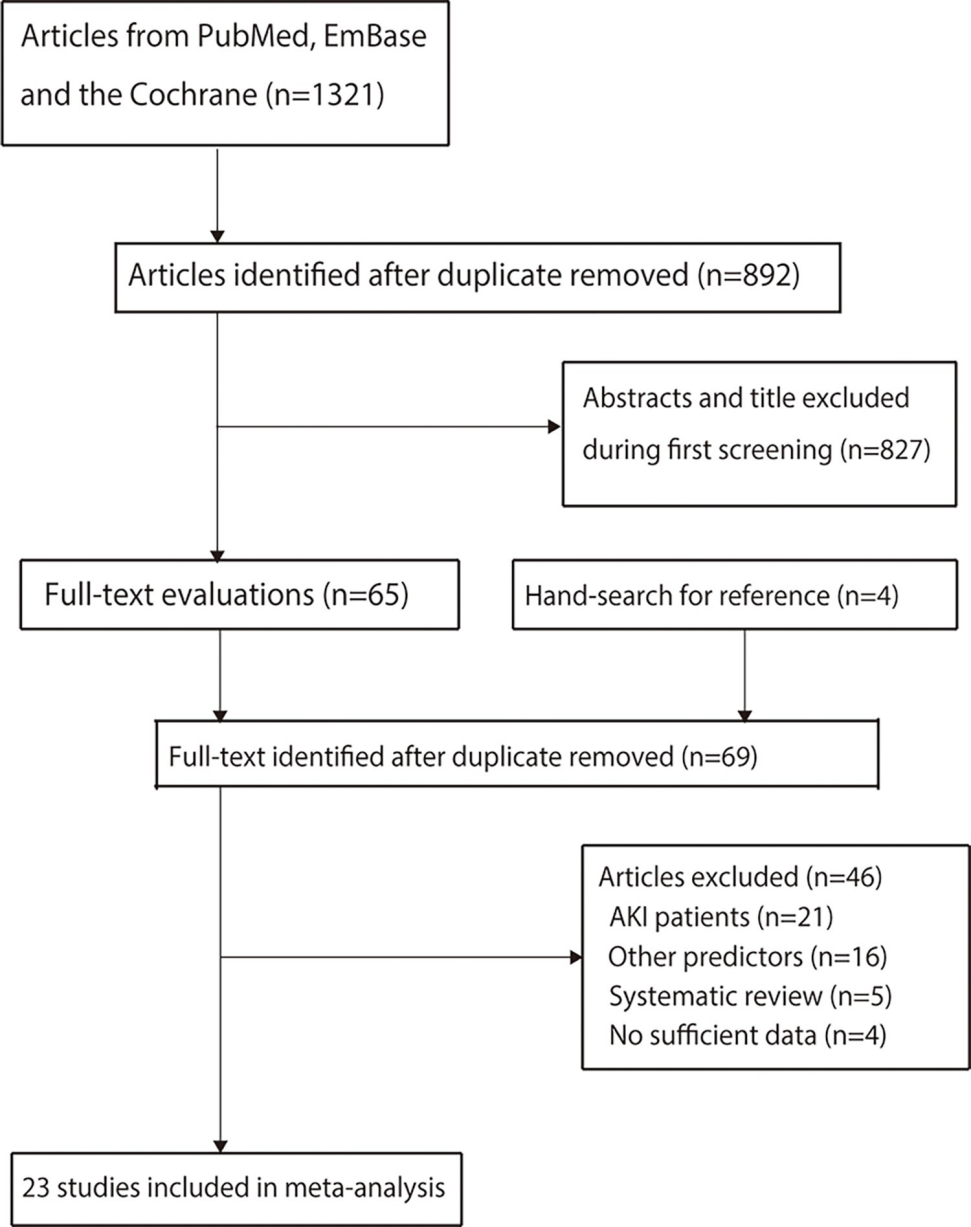
Flow diagram of the literature search and studies selection process.

The process of literature search and study selection are shown in Fig 1 and S1 Flowchart. An initial electronic search yielded 1,321 articles, among which 892 studies were retained after removing duplicates. Another 827 studies were removed since they reported irrelevant titles or contents. The remaining 65 studies were retrieved for full-text evaluations, and 42 studies were excluded because of the presence of AKI patients (n = 21), other predictors (n = 16), and being systematic reviews (n = 5). By reviewing the list of references, we found 4 candidate studies; however, they were removed due to lack of sufficient data. Finally, a total of 23 prospective studies were included in this meta-analysis, and included studies are listed in S1 Appendix.

### Study characteristics

S1 Table summarizes the characteristics of the included studies and the involved patients. All of the included studies were published between 2011 and 2021, and the number of patients in each study ranged from 48–371 patients. Twenty-one studies included patients in the intensive care unit, while 2 other studies included patients at the ward. Regarding the country, 9 studies were conducted in France, 6 studies were conducted in China, 2 studies were performed in the Netherlands, and 1 study was each performed in Germany, Turkey, Poland, Sweden, Italy, and USA. All of the studies reported the predictive performance of RRI for AKI, while only 5 studies reported the predictive performance of PDU for AKI. The quality assessment in each individual study is shown in S2 Table.

### Sensitivity and specificity

The sensitivity and specificity of the RRI and PDU for predicting AKI are shown in S1 Fig. The pooled sensitivity and specificity were 0.76 (95% CI: 0.70–0.81; *I*^*2*^ = 80.33%; *P* < 0.01) and 0.79 (95% CI: 0.72–0.85; *I*^*2*^ = 94.40%; *P* < 0.01) for the RRI, and 0.64 (95% CI: 0.48–0.77; *I*^*2*^ = 90.85%; *P* < 0.01) and 0.90 (95%: 0.81–0.95; *I*^*2*^ = 93.17%; *P*<0.01) for the PDU, respectively. We noted that the sensitivity between the RRI and PDU were not significantly different (ratio: 1.19; 95% CI: 0.93–1.52; P = 0.165), while the RRI showed a lower specificity for predicting AKI than the PDU (ratio: 0.88; 95% CI: 0.78–0.98; P = 0.028). The subgroup analyses for the pooled studies indicated that the RRI had a higher sensitivity compared to PDU for Western countries, mean age of patients <65.0 years, male proportion ≥60.0%, and patients with comorbidities other than having undergone cardiothoracic surgery. The results also demonstrated that the country, mean age, and disease status could affect the predictive performance of the PDU for AKI risk. Furthermore, the RRI was associated with a lower specificity than the PDU when the studies were conducted in Eastern countries, mean age of patients ≥65.0 years, and male proportion <60.0%. The predictive performance of the PDU for AKI could be affected by the country, mean age, male proportion, and disease status of patients (Table 1).

**Table 1:** Subgroup analyses for diagnostic parameters.

Parameters	Variables	Subgroups	Index	Effect estimates and 95%CI	*I*^*2*^ *(%)*	Q statistic	Ratio between indexes	Ratio for RRI between subgroups	Ratio for PDU between subgroups
Sensitivity	Country	Eastern	RRI	0.74 (0.67–0.79)	0.0	0.51	1.10 (0.92–1.32)/ 0.301	0.96 (0.85–1.09)/ 0.520	1.52 (1.19–1.94)/ 0.001
			PDU	0.67 (0.56–0.77)	23.0	0.27			
		Western	RRI	0.77 (0.69–0.84)	86.8	< 0.01	1.75 (1.42–2.16)/< 0.001		
			PDU	0.44 (0.36–0.52)	90.40	< 0.01			
	Age (years)	≥ 65.0	RRI	0.75 (0.68–0.81)	58.58	< 0.01	1.12 (0.93–1.34)/ 0.224	0.97 (0.84–1.14)/ 0.696	1.52 (1.19–1.94)/ 0.001
			PDU	0.67 (0.56–0.77)	23.0	0.27			
		< 65.0	RRI	0.77 (0.66–0.85)	93.10	< 0.01	1.75 (1.40–2.19)/ < 0.001		
			PDU	0.44 (0.36–0.52)	90.40	< 0.01			
	Male (%)	≥ 60.0	RRI	0.76 (0.69–0.81)	84.58	< 0.01	1.58 (1.32–1.89)/ < 0.001	1.00 (0.86–1.16)/ 1.000	0.72 (0.56–0.92)/ 0.009
			PDU	0.48 (0.40–0.55)	88.30	< 0.01			
		< 60.0	RRI	0.76 (0.65–0.84)	46.13	0.12	1.13 (0.90–1.43)/ 0.301		
			PDU	0.67 (0.54–0.79)	18.70	0.27			
	Disease status	Cardiothoracic surgery	RRI	0.77 (0.70–0.83)	53.28	0.01	1.22 (0.99–1.51)/ 0.065	1.04 (0.91–1.20)/ 0.578	1.31 (1.02–1.69)/ 0.036
			PDU	0.63 (0.50–0.74)	0.00	0.92			
		Other	RRI	0.74 (0.65–0.81)	86.60	< 0.01	1.54 (1.27–1.87)/ < 0.001		
			PDU	0.48 (0.41–0.56)	90.60	< 0.01			
Specificity	Country	Eastern	RRI	0.77 (0.64–0.86)	91.94	< 0.01	0.86 (0.73–1.00)/ 0.060	0.96 (0.80–1.15)/ 0.659	1.14 (1.05–1.23)/ 0.001
			PDU	0.90 (0.85–0.93)	0.00	0.78			
		Western	RRI	0.80 (0.71–0.87)	96.04	< 0.01	1.01 (0.90–1.14)/ 0.869		
			PDU	0.79 (0.74–0.84)	96.00	< 0.01			
	Age (years)	≥ 65.0	RRI	0.78 (0.70–0.84)	85.13	< 0.01	0.87 (0.78–0.96)/ 0.009	0.94 (0.78–1.13)/ 0.513	1.14 (1.05–1.23)/ 0.001
			PDU	0.90 (0.85–0.93)	0.00	0.78			
		< 65.0	RRI	0.83 (0.67–0.92)	97.83	< 0.01	1.05 (0.89–1.25)/ 0.573		
			PDU	0.79 (0.74–0.84)	96.00	< 0.01			
	Male (%)	≥ 60.0	RRI	0.80 (0.71–0.86)	95.48	< 0.01	0.99 (0.89–1.10)/ 0.852	1.04 (0.90–1.20)/ 0.593	0.88 (0.82–0.95)/ 0.01
			PDU	0.81 (0.77–0.85)	86.80	< 0.01			
		< 60.0	RRI	0.77 (0.68–0.84)	70.45	0.01	0.84 (0.74–0.94)/ 0.004		
			PDU	0.92 (0.86–0.96)	89.80	< 0.01			
	Disease status	Cardiothoracic surgery	RRI	0.81 (0.72–0.88)	87.03	< 0.01	0.91 (0.81–1.02)/ 0.109	1.05 (0.88–1.26)/ 0.594	1.09 (1.01–1.17)/ 0.022
			PDU	0.89 (0.83–0.93)	0.00	0.66			
		Other	RRI	0.77 (0.64–0.86)	96.06	< 0.01	0.94 (0.80–1.10)/ 0.446		
			PDU	0.82 (0.78–0.86)	93.60	< 0.01			
PLR	Country	Eastern	RRI	3.17 (1.95–5.14)	65.75	< 0.01	0.48 (0.26–0.90)/ 0.021	0.81 (0.43–1.55)/ 0.519	0.75 (0.01–56.71)/ 0.896
			PDU	6.57 (4.40–9.81)	0.00	0.50			
		Western	RRI	3.90 (2.56–5.95)	95.04	< 0.01	0.44 (0.01–33.73)/ 0.692		
			PDU	8.81 (0.12–668.56)	89.8	< 0.01			
	Age (years)	≥ 65.0	RRI	3.35 (2.44–4.60)	76.19	< 0.01	0.51 (0.31–0.85)/ 0.009	0.75 (0.32–1.74)/ 0.505	0.75 (0.01–56.71)/ 0.896
			PDU	6.57 (4.40–9.81)	0.00	0.50			
		< 65.0	RRI	4.49 (2.05–9.82)	97.37	< 0.01	0.51 (0.01–40.82)/ 0.751		
			PDU	8.81 (0.12–668.56)	89.8	< 0.01			
	Male (%)	≥ 60.0	RRI	3.71 (2.44–5.63)	94.44	< 0.01	0.84 (0.22–3.28)/ 0.800	1.13 (0.67–1.90)/ 0.646	0.29 (0.01–7.95)/ 0.467
			PDU	4.41 (1.21–16.10)	92.20	< 0.01			
		< 60.0	RRI	3.29 (2.42–4.48)	0.00	0.18	0.21 (0.01–4.64)/ 0.319		
			PDU	15.41 (0.72–329.21)	79.50	0.03			
	Disease status	Cardiothoracic surgery	RRI	4.10 (2.65–6.34)	79.84	< 0.01	0.72 (0.38–1.38)/ 0.318	1.29 (0.68–2.48)/ 0.440	0.83 (0.13–5.18)/ 0.843
			PDU	5.69 (3.53–9.17)	0.00	0.65			
		Other	RRI	3.17 (1.96–5.14)	94.49	< 0.01	0.46 (0.07–2.89)/ 0.413		
			PDU	6.82 (1.17–39.72)	92.60	< 0.01			
NLR	Country	Eastern	RRI	0.34 (0.26–0.45)	29.91	0.20	0.89 (0.59–1.36)/ 0.584	1.21 (0.78–1.89)/ 0.399	0.79 (0.22–2.81)/ 0.717
			PDU	0.38 (0.28–0.53)	13.50	0.31			
		Western	RRI	0.28 (0.20–0.40)	92.24	< 0.01	0.58 (0.16–2.09)/ 0.406		
			PDU	0.48 (0.14–1.63)	91.0	< 0.01			
	Age (years)	≥ 65.0	RRI	0.32 (0.25–0.42)	57.93	< 0.01	0.84 (0.56–1.27)/ 0.404	1.14 (0.68–1.93)/ 0.622	0.79 (0.22–2.81)/ 0.717
			PDU	0.38 (0.28–0.53)	13.50	0.31			
		< 65.0	RRI	0.28 (0.18–0.45)	96.41	< 0.01	0.58 (0.16–2.16)/ 0.412		
			PDU	0.48 (0.14–1.63)	91.0	< 0.01			
	Male (%)	≥ 60.0	RRI	0.31 (0.23–0.41)	91.06	< 0.01	0.69 (0.29–1.62)/ 0.398	1.00 (0.63–1.58)/ 1.000	1.25 (0.49–3.21)/ 0.642
			PDU	0.45 (0.20–1.00)	89.40	< 0.01			
		< 60.0	RRI	0.31 (0.22–0.45)	20.62	0.28	0.86 (0.47–1.58)/ 0.626		
			PDU	0.36 (0.22–0.59)	36.60	0.21			
	Disease status	Cardiothoracic surgery	RRI	0.28 (0.20–0.40)	64.19	< 0.01	0.67 (0.42–1.06)/ 0.090	0.82 (0.50–1.34)/ 0.430	1.14 (0.35–3.67)/ 0.827
			PDU	0.42 (0.31–0.57)	0.00	0.86			
		Other	RRI	0.34 (0.24–0.48)	91.87	< 0.01	0.92 (0.28–3.01)/ 0.891		
			PDU	0.37 (0.12–1.16)	91.40	< 0.01			
DOR	Country	Eastern	RRI	8.01 (4.19–15.34)	57.00	0.03	0.44 (0.17–1.14)/ 0.091	0.57 (0.21–1.54)/ 0.269	0.95 (0.01–175.56)/ 0.984
			PDU	18.30 (9.05–36.99)	18.50	0.29			
		Western	RRI	13.98 (6.62–29.52)	86.50	< 0.01	0.72 (0.00–134.93)/ 0.913		
			PDU	19.32 (0.11–3435.07)	91.80	< 0.01			
	Age (years)	≥ 65.0	RRI	9.81 (5.92–16.27)	61.90	< 0.01	0.54 (0.23–1.28)/ 0.159	0.66 (0.19–2.28)/ 0.512	0.95 (0.01–175.56)/ 0.984
			PDU	18.30 (9.05–36.99)	18.50	0.29			
		< 65.0	RRI	14.89 (4.79–46.25)	90.10	< 0.01	0.77 (0.00–153.98)/ 0.932		
			PDU	19.32 (0.11–3435.07)	91.80	< 0.01			
	Male (%)	≥ 60.0	RRI	11.71 (6.05–22.64)	85.20	< 0.01	1.12 (0.13–9.85)/ 0.918	1.13 (0.49–2.62)/ 0.775	0.23 (0.01–10.37)/ 0.407
			PDU	10.43 (1.32–82.69)	92.60	< 0.01			
		< 60.0	RRI	10.32 (6.17–17.26)	0.00	0.52	0.23 (0.01–5.78)/ 0.365		
			PDU	44.53 (1.86–1066.67)	77.40	0.04			
	Disease status	Cardiothoracic surgery	RRI	13.77 (6.94–27.35)	68.40	<0.01	1.00 (0.37–2.68)/ 1.000	1.46 (0.51–4.17)/ 0.480	0.60 (0.03–12.43)/ 0.740
			PDU	13.74 (6.78–27.86)	0.00	0.70			
		Other	RRI	9.42 (4.27–20.81)	86.7	< 0.01	0.41 (0.02–8.70)/ 0.565		
			PDU	22.81 (1.20–432.06)	93.20	< 0.01			
AUC	Country	Eastern	RRI	0.75 (0.71–0.78)	-	-	0.77 (0.69–0.86)/ < 0.001	0.88 (0.83–0.94)/ < 0.001	-
			PDU	0.97 (0.81–0.99)	-	-			
		Western	RRI	0.85 (0.82–0.88)	-	-			
			PDU	-	-	-			
	Age (years)	≥ 65.0	RRI	0.82 (0.79–0.85)	-	-	0.85 (0.76–0.94)/ 0.003	0.96 (0.91–1.02)/ 0.161	-
			PDU	0.97 (0.81–0.99)	-	-			
		< 65.0	RRI	0.85 (0.81–0.88)	-	-	-		
			PDU	-	-	-			
	Male (%)	≥ 60.0	RRI	0.83 (0.80–0.86)	-	-	0.85 (0.77–0.93)/ 0.001	1.00 (0.95–1.05)/ 1.000	-
			PDU	0.98 (0.85–1.00)	-	-			
		< 60.0	RRI	0.83 (0.80–0.86)	-	-	-		
			PDU	-	-	-			
	Disease status	Cardiothoracic surgery	RRI	0.85 (0.82–0.88)	-	-	-	1.05 (0.99–1.11)/ 0.095	-
			PDU	-	-	-			
		Other	RRI	0.81 (0.77–0.84)	-	-	1.21 (0.04–38.24)/ 0.913		
			PDU	0.67 (0.00–1.00)	-	-			

### PLR and NLR

The summary of the PLR and NLR of the RRI and PDU for predicting AKI are shown in S2 Fig. We noted that the pooled PLR and NLR for predicting AKI were 3.64 (95% CI: 2.61–5.08; *I*^*2*^ = 92.98%; *P* < 0.01) and 0.31 (95% CI: 0.24–0.39; *I*^*2*^ = 88.19%; *P* < 0.01), and 6.58 (95% CI: 2.79–15.52; *I*^*2*^ = 92.40%; *P* < 0.01) and 0.40 (95% CI: 0.25–0.64; *I*^*2*^ = 95.54%; *P* < 0.01) for the RRI and PDU, respectively. There were no significant differences between the RRI and PDU for the PLR (ratio: 0.55; 95% CI: 0.22–1.39; P = 0.204) and NLR (ratio: 0.77; 95% CI: 0.46–1.32; P = 0.331). The subgroup analyses showed that the RRI had a lower PLR than the PDU if the studies were conducted in Eastern countries and the mean age of patients was ≥65.0 years, while no significant difference was observed in the NLR between the RRI and PDU in any subgroup. Moreover, the predictive performance of the RRI and PDU for AKI was not affected by the country, mean age, male proportion, and disease status (Table 1).

### DOR

The summary of the DOR of the RRI and PDU for predicting AKI are shown in S3 Fig. We noted that the pooled DOR for the RRI and PDU were 11.76 (95% CI: 6.76–20.45), and 16.32 (95% CI: 3.60–74.07), respectively, while significant heterogeneity for the RRI (*I*^*2*^ = 82.90%; *P* < 0.01) and PDU (*I*^*2*^ = 90.50%; *P* < 0.01) were observed. No significant difference between the RRI and PDU for the DOR was detected (ratio: 0.72; 95% CI: 0.14–3.61; P = 0.692). The subgroup analyses showed that the difference between the RRI and PDU for the DOR were not statistically significant in all subgroups, while the role of the RRI and PDU on the DOR were not affected by the country, mean age, male proportion, and disease status (Table 1).

### AUC

**Fig 2:**
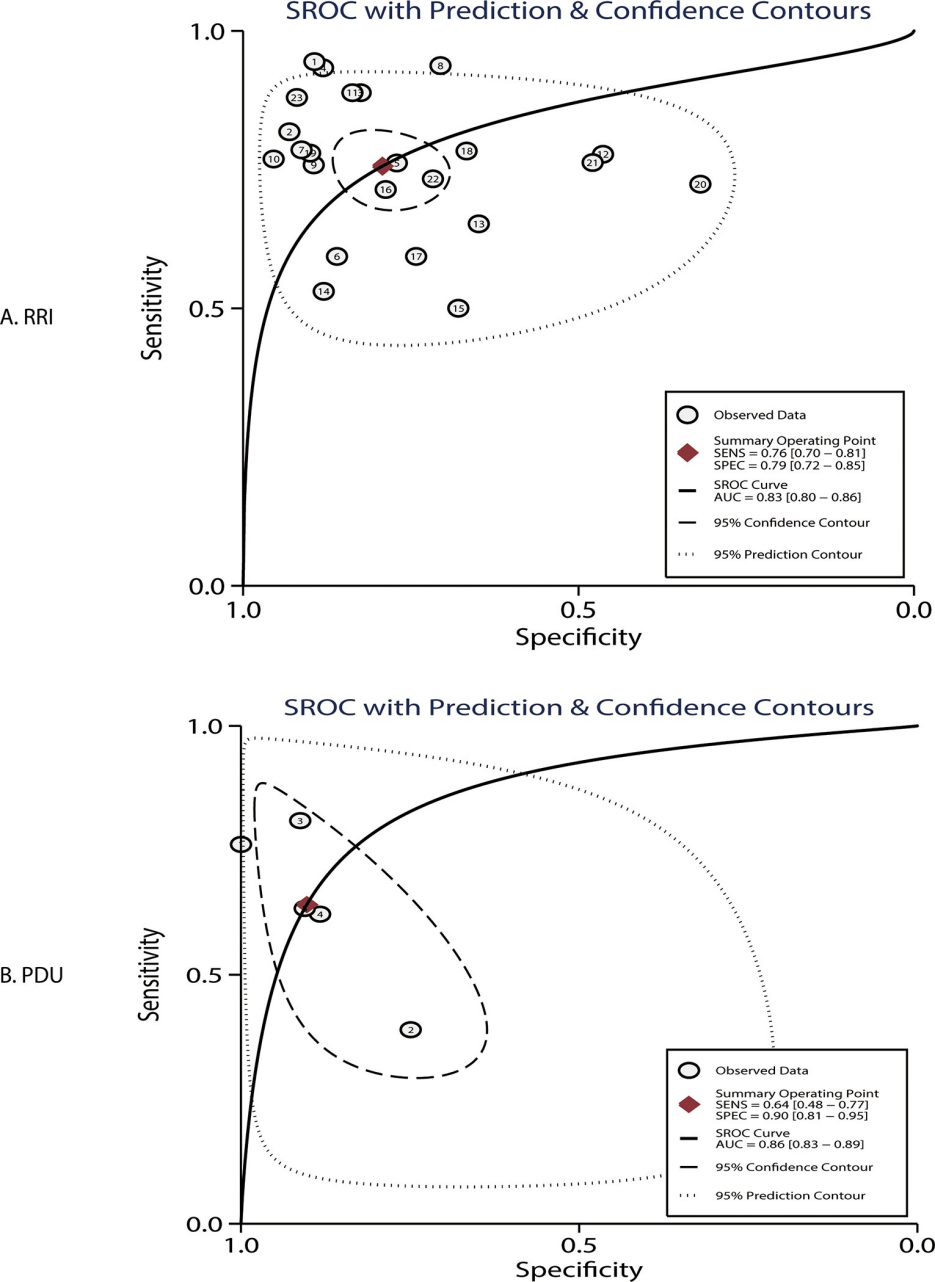
Summary of the AUC of the RRI and PDU for predicting AKI.

The summary of the AUC of the RRI and PDU for predicting AKI is shown in Fig 2. The pooled AUCs for the RRI and PDU were 0.83 (95% CI: 0.80–0.86) and 0.86 (95% CI: 0.83–0.89), respectively. Moreover, there was no significant difference between the RRI and PDU for the AUC (ratio: 0.97; 95% CI: 0.92–1.01; P = 0.201). For the subgroup analyses, the RRI showed a lower AUC than the PDU if the studies were conducted in Eastern countries, mean age of patients ≥65.0 years, and male proportion ≥60.0%. The AUC of the RRI in Eastern countries was lower than that in Western countries (Table 1).

### Publication bias

**Fig 3:**
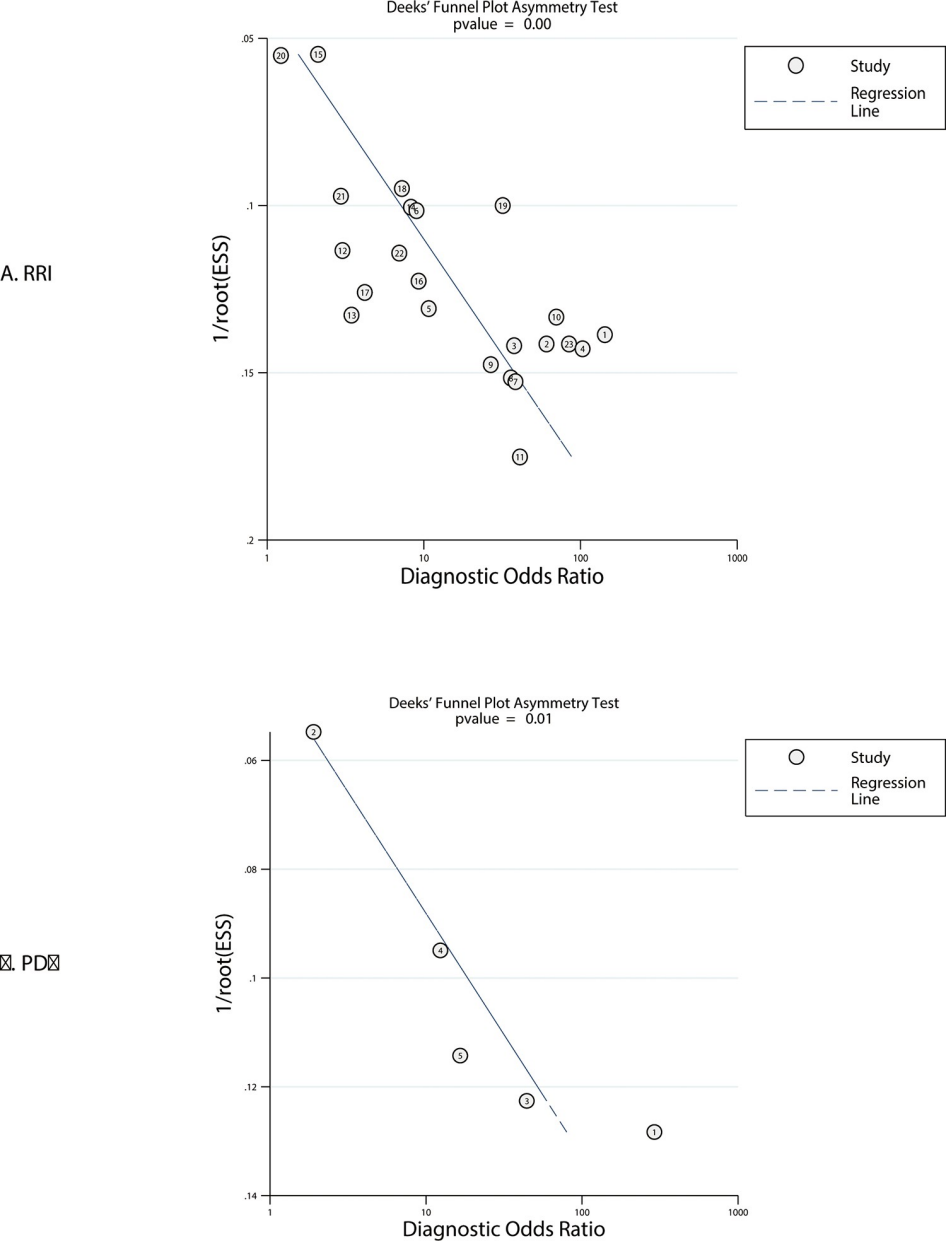
Funnel plots for the predictive performance of the RRI and PDU.

Publication biases for the RRI and PDU were assessed in Fig 3. There was potential significant publication bias of the RRI (*P* < 0.01) and PDU (*P* = 0.01) for predicting AKI. However, after adjusting for the potential publication bias using the trim and fill method, the DOR for the RRI and PDU was calculated at 32.22 (95% CI: 22.88–41.56; *P* < 0.001) and 73.20 (95% CI: 29.54–116.86; *P* = 0.001), respectively (S4 Fig).

## Discussion

Nowadays, numerous studies have constructed prediction model for AKI in patients undergoing various conditions, and the model were constructed based on perioperative laboratory test and history of disease status [22–27]. However, the prediction model for AKI were not contained the parameters from ultrasound. Therefore, this systematic review and meta-analysis was performed on prospective studies and assessed the predictive performance of the RRI and PDU for predicting AKI. A total of 2,400 patients from 23 prospective studies were included, and the patients had a broad range of characteristics. This study showed that the predictive performance of the RRI and PDU on subsequent AKI was comparable, and they had similar sensitivities, PLRs, NLRs, DORs, and AUCs. However, the specificity of the RRI was significantly lower than that of the PDU for predicting the AKI risk. The subgroup analyses found that the sensitivity of the RRI was higher than that of the PDU if the pooled studies were conducted in Western countries, mean age of patients <65.0 years, male proportion ≥60.0%, and patients had comorbidities other than having undergone cardiothoracic surgery, whereas the specificity of the RRI was lower than that of the PDU if the pooled studies were conducted in Eastern countries, mean age of patients ≥65.0 years, and male proportion <60.0%. Moreover, the RRI had a lower PLR than the PDU if the pooled studies were conducted in Eastern countries and the mean age of patients ≥65.0 years. Finally, we noted that the RRI had a lower AUC as compared with the PDU if the pooled studies were conducted in Eastern countries, mean age of patients ≥65.0 years, and male proportion ≥60.0%.

Several systematic reviews and meta-analyses have already investigated the predictive performance of the RRI for subsequent AKI risk [28–30]. Ninet et al. performed a meta-analysis of 9 studies and found that an elevated RRI could predict persistent AKI in critically ill patients [28]. Bellos et al. identified 10 studies and found that the RRI could be considered as an important marker for predicting of postoperative AKI [29]. Wu et al. conducted a meta-analysis of 20 studies and found that an elevated RRI was significantly related to the onset of AKI, and the predictive performance of the RRI for AKI and short-term non-recovery was relative good [30]. However, several limitations of prior studies should be mentioned: (1) the predictive performance of the RRI according to the studies or patients’ characteristics were not illustrated; (2) the predictive performance of the PDU for the risk of AKI was not determined; and (3) the predictive performance of the RRI and PDU for AKI in patients with critical illnesses were not compared. Therefore, this study was performed to assess the predictive performance of the RRI and PDU for subsequent AKI risk. Moreover, an indirect comparison for the predictive performance of RRI and PDU and subsequent AKI risk was also performed.

This study found that the predictive performance of the RRI and PDU are similar, while the specificity of the RRI was lower than that of PDU for predicting AKI risk. The RRI could be affected by several pathological conditions, including hypovolemia, rhabdomyolysis, sepsis, nephrotoxic substances, and multiple organ failure [31]. This phase indicated that the RRI could not predict AKI at early stages, owing to it was not the determined progression of AKI. Moreover, the RRI should be considered as a marker for kidney damage and renal function, and its predictive role on the subsequent AKI risk could be affected by several factors. Moreover, although the PDU has a higher specificity for predicting AKI than RRI, the PDU score has the following limitations: it presents significant soft-tissue flash artifacts, and breathing movements could affect the PDU results. Moreover, the imaging results of the PDU could be altered in obese patients and is dependent on the physician’s skill.

The subgroup analysis found that the RRI had higher sensitivity than the PDU when the pooled studies were conducted in Western countries, mean age of patients <65.0 years, male proportion ≥60.0%, and patients had other comorbidities. Moreover, the specificity of the RRI and PDU for predicting AKI are similar in these subgroups. These results suggest that the RRI is superior to the PDU for predicting AKI in Western countries, younger patients, males, and patients not treated for cardiothoracic surgery. Several reasons could have led to these results: (1) the different skill and training levels of radiologists between Eastern and Western countries [32]; (2) the age of patients could have affected the severity and prognosis of AKI; (3) the risk stratification between male and female were different, which could have affected the prevalence or severity of AKI; and (4) the pathophysiological mechanisms of AKI in patients undergoing various conditions were different. One example is the progression of AKI in patients undergoing cardiac failure, which include hemodynamic, neurohormonal, inflammatory, and oxidative stress-related mechanisms, while the renal vasculature and results in sluggish flow, congestion or glomerular dysfunction significantly affect the progression of AKI [33]. Finally, the stratified analyses for the predictive performance of the PDU was based on a smaller number of studies, which caused the results of the indirect comparison in the subgroup analyses to be variable.

The limitations of this study should be acknowledged: (1) the baseline characteristics of patients varied, and a significant heterogeneity for predictive performance was observed, which was not fully explained using the subgroup analyses; (2) the definitions of AKI across the included studies were varied, which included transient and persistent AKI; (3) the predictive performance of the PDU was available in only a small number of the included studies; thus, the results of indirect comparison in the stratified analyses were very limited; and (4) the presence of inherent limitations for the meta-analysis on the basis of published articles, including inevitable publication bias and restricted detailed analyses.

## Conclusions

This study showed that the predictive parameters of the RRI and PDU for subsequent AKI risk are comparable, while the specificity of the PDU was higher than that of the RRI for predicting AKI risk. The subgroup analyses found that the predictive performance of the RRI was superior to that of the PDU when the pooled studies were conducted in Western countries, mean age of patients were <65.0 years, male proportion was ≥60.0%, and patients had comorbidities other than cardiothoracic surgery, which suggested that the RRI should be applied in these subpopulations. Further large-scale prospective studies should be performed to direct comparison the prediction performance of RRI and PDU for subsequent AKI risk in patients with various medical conditions. Moreover, the prediction model for AKI risk should be updated and contained indexes from ultrasound using machine learning approach.

## Supporting information

S1 Checklist PRISMA checklist. (DOCX)

S1 Flowchart PRISMA flowchart. (DOCX)

S1 Appendix The details of included studies. (DOCX)

S1 File The details of abstracted data. (XLSX)

S1 Table The characteristics of included studies. (DOCX)

S2 Table The quality of methodological assessed using quality assessment of diagnostic accuracy studies 2 scoring system. (DOCX)

S1 Fig Summary of the sensitivity and specificity of the RRI and PDU for predicting AKI. (TIF)

S2 Fig Summary of the PLR and NLR of the RRI and PDU for predicting AKI. (TIF)

S3 Fig Summary of the DOR of the RRI and PDU for predicting AKI. (TIF)

S4 Fig Adjusted publication bias using the trim and fill method. (TIF)

## Abbreviations

AKI: Acute kidney injury
AUC: Area under the receiver operating characteristic curves
CI: Confidence interval
DOR: Diagnostic odds ratio
NLR: Negative likelihood ratios
PDU: Power Doppler ultrasound
PLA: Positive likelihood ratios
RRI: Renal Resistive Index
