# Correction: Performance of resistive index and semi-quantitative power doppler ultrasound score in predicting acute kidney injury: A meta-analysis of prospective studies

**DOI:** 10.1371/journal.pone.0315513

**Published:** 2024-12-05

**Authors:** Yu Zhu, Weifeng Zhen, Xiaoning Zhang, Zhenhua Shi, Ling Zhang, Jiuju Zhou, Qiong Wei

The authors are listed out of order. Please view the correct author order and citation here:

Yu Zhu, Weifeng Zhen, Xiaoning Zhang, Zhenhua Shi, Ling Zhang, Jiuju Zhou, Qiong Wei

Zhu Y, Zhen W, Zhang X, Shi Z, Zhang L, Zhou J, et al. (2022) Performance of resistive index and semi-quantitative power doppler ultrasound score in predicting acute kidney injury: A meta-analysis of prospective studies. PLOS ONE 17(6): e0270623. https://doi.org/10.1371/journal.pone.0270623
