# Supplementary material for: Performance of resistive index and semi-quantitative power doppler ultrasound score in predicting acute kidney injury: A meta-analysis of prospective studies
Source: PLoS One. 2022 Jun 28;17(6):e0270623. doi: 10.1371/journal.pone.0270623 (PMC9239473; doi:10.1371/journal.pone.0270623)
Supplement: S1 Appendix — (DOCX) [file pone.0270623.s003.docx]

**Reference**

1. Bossard G, Bourgoin P, Corbeau JJ, *et al*. Early detection of postoperative acute kidney injury by Doppler renal resistive index in cardiac surgery with cardiopulmonary bypass. *Br J Anaesth* 2011;107:891-8.
2. Darmon M, Schortgen F, Vargas F, *et al*. Diagnostic accuracy of Doppler renal resistive index for reversibility of acute kidney injury in critically ill patients. *Intensive Care Med* 2011;37:68-76.
3. Schnell D, Deruddre S, Harrois A, *et al*. Renal resistive index better predicts the occurrence of acute kidney injury than cystatin C. *Shock* 2012;38:592-7.
4. Guinot PG, Bernard E, Abou Arab O, *et al*. Doppler-based renal resistive index can assess progression of acute kidney injury in patients undergoing cardiac surgery. *J Cardiothorac Vasc Anesth* 2013;27:890-6.
5. Schnell D, Reynaud M, Venot M, *et al*. Resistive Index or color-Doppler semi-quantitative evaluation of renal perfusion by inexperienced physicians: results of a pilot study. *Minerva Anestesiol* 2014;80:1273-81.
6. Sinning JM, Adenauer V, Scheer AC, *et al*. Doppler-based renal resistance index for the detection of acute kidney injury and the non-invasive evaluation of paravalvular aortic regurgitation after transcatheter aortic valve implantation. *EuroIntervention* 2014;9:1309-16.
7. Kararmaz A, Kemal Arslantas M, Cinel I. Renal Resistive Index Measurement by Transesophageal Echocardiography: Comparison With Translumbar Ultrasonography and Relation to Acute Kidney Injury. *J Cardiothorac Vasc Anesth* 2015;29:875-80.
8. Marty P, Szatjnic S, Ferre F, *et al*. Doppler renal resistive index for early detection of acute kidney injury after major orthopaedic surgery: a prospective observational study. *Eur J Anaesthesiol* 2015;32:37-43.
9. Marty P, Ferre F, Labaste F, *et al*. The Doppler renal resistive index for early detection of acute kidney injury after hip fracture. *Anaesth Crit Care Pain Med* 2016;35:377-382.
10. Qin H, Wu H, Chen Y, *et al*. Early Detection of Postoperative Acute Kidney Injury in Acute Stanford Type A Aortic Dissection With Doppler Renal Resistive Index. *J Ultrasound Med* 2017;36:2105-2111.
11. Wybraniec MT, Chudek J, Bożentowicz-Wikarek M, *et al*. Prediction of contrast-induced acute kidney injury by early post-procedural analysis of urinary biomarkers and intra-renal Doppler flow indices in patients undergoing coronary angiography. *J Interv Cardiol* 2017;30:465-472.
12. Hertzberg D, Ceder SL, Sartipy U, *et al*. Preoperative Renal Resistive Index Predicts Risk of Acute Kidney Injury in Patients Undergoing Cardiac Surgery. *J Cardiothorac Vasc Anesth* 2017;31:847-852.
13. Regolisti G, Maggiore U, Cademartiri C, *et al*. Renal resistive index by transesophageal and transparietal echo-doppler imaging for the prediction of acute kidney injury in patients undergoing major heart surgery. *J Nephrol* 2017;30:243-253.
14. Haitsma Mulier JLG, Rozemeijer S, Röttgering JG, *et al*. Renal resistive index as an early predictor and discriminator of acute kidney injury in critically ill patients; A prospective observational cohort study. *PLoS One* 2018;13:e0197967.
15. Darmon M, Bourmaud A, Reynaud M, *et al*. Performance of Doppler-based resistive index and semi-quantitative renal perfusion in predicting persistent AKI: results of a prospective multicenter study. *Intensive Care Med* 2018;44:1904-1913.
16. Zhi HJ, Zhao J, Nie S, *et al*. Prediction of acute kidney injury: the ratio of renal resistive index to semiquantitative power Doppler ultrasound score-a better predictor?: A prospective observational study. *Medicine (Baltimore)* 2019;98:e15465.
17. Zhi HJ, Zhang M, Cui XY, *et al*. Renal echography and cystatin C for prediction of acute kidney injury: very different in patients with cardiac failure or sepsis. *Chin Crit Care Med* 2019; 31: 1258-1263.
18. Zhi HJ, Li Y, Wang B, *et al*. Renal echography for predicting acute kidney injury in critically ill patients: a prospective observational study. *Ren Fail* 2020;42:263-269.
19. Garnier F, Daubin D, Larcher R, *et al*. Reversibility of Acute Kidney Injury in Medical ICU Patients: Predictability Performance of Urinary Tissue Inhibitor of Metalloproteinase-2 x Insulin-Like Growth Factor-Binding Protein 7 and Renal Resistive Index. *Crit Care Med* 2020; 48:e277-e284.
20. Wiersema R, Kaufmann T, van der Veen HN, *et al*. Diagnostic accuracy of arterial and venous renal Doppler assessment for acute kidney injury in critically ill patients: A prospective study. *J Crit Care* 2020;59:57-62.
21. Fu Y, He C, Bai Y, *et al*. Value of the combination of renal resistive index and central venous pressure to predict septic shock induced acute kidney injury. *Zhonghua Wei Zhong Bing Ji Jiu Yi Xue* 2020;32:473-477.
22. Zhi HJ, Zhao J, Nie S, *et al*. Semiquantitative Power Doppler Ultrasound Score to Predict Acute Kidney Injury in Patients With Sepsis or Cardiac Failure: A Prospective Observational Study. *J Intensive Care Med* 2021;36:115-122.

Shankar V, Raj A, Singhal S, *et al*. Doppler-derived renal resistive index helps predict acute kidney injury in patients undergoing living-related liver transplantation. *Clin Transplant* 2021;35:e14263.
