# Supplementary material for: Performance of resistive index and semi-quantitative power doppler ultrasound score in predicting acute kidney injury: A meta-analysis of prospective studies
Source: PLoS One. 2022 Jun 28;17(6):e0270623. doi: 10.1371/journal.pone.0270623 (PMC9239473; doi:10.1371/journal.pone.0270623)
Supplement: S1 Table — (DOCX) [file pone.0270623.s005.docx]

S1 Table. The characteristics of included studies

| Study | Country | Setting | Sample size | Age (years) | Male (%) | Disease status | Index and cutoff value | AKI (n, %) | TP | FP | FN | TN |
| --- | --- | --- | --- | --- | --- | --- | --- | --- | --- | --- | --- | --- |
| Bossard 2011 [1] | France | ICU | 65 | 74.8 | 67.7 | Cardiac surgery | RRI (0.740) | 18 (27.7) | 17 | 5 | 1 | 42 |
| Darmon 2011 [2] | France | ICU | 51 | 62.0 | 73.0 | MV | RRI (0.795) | 22 (43.1) | 18 | 2 | 4 | 27 |
| Schnell 2012 [3] | France | ICU | 58 | 44.0 | 69.0 | Critically illness | RRI (0.707) | 18 (31.0) | 16 | 7 | 2 | 33 |
| Guinot 2013 [4] | France | ICU | 82 | 69.0 | 70.7 | Cardiac surgery | RRI (0.730) | 15 (18.3) | 14 | 8 | 1 | 59 |
| Schnell 2014 [5] | France | ICU | 69 | 57.0 | 57.0 | MV | RRI (0.750) | 21 (30.4) | 16 | 11 | 5 | 37 |
|  |  |  |  |  |  |  | Semi-quantitative PDU (1.0) |  | 16 | 0 | 5 | 48 |
| Sinning 2014 [6] | Germany | ICU | 132 | 80.9 | 53.8 | TAVI | RRI (0.850) | 32 (24.2) | 19 | 14 | 13 | 86 |
| Kararmaz 2015 [7] | Turkey | ICU | 60 | 58.6 | 68.3 | Cardiac surgery | RRI (0.730) | 14 (23.3) | 11 | 4 | 3 | 42 |
| Marty 2015 [8] | France | Ward | 50 | 72.0 | 42.0 | Major orthopaedic surgery | RRI (0.705) | 16 (32.0) | 15 | 10 | 1 | 24 |
| Marty 2016 [9] | France | Ward | 48 | 83.0 | 43.8 | Hip fracture surgery | RRI (0.706) | 29 (60.4) | 22 | 2 | 7 | 17 |
| Qin 2017 [10] | China | ICU | 61 | 47.2 | 86.9 | AAAD | RRI (0.710) | 39 (63.9) | 30 | 1 | 9 | 21 |
| Wybraniec 2017 [11] | Poland | ICU | 95 | 65.0 | 69.5 | Cardiac surgery | RRI (0.730) | 9 (9.5) | 8 | 14 | 1 | 72 |
| Hertzberg 2017 [12] | Sweden | ICU | 96 | 69.0 | 72.9 | Cardiac surgery | RRI (0.700) | 27 (28.1) | 21 | 37 | 6 | 32 |
| Regolisti 2017 [13] | Italy | ICU | 60 | 69.5 | 68.3 | Cardiac surgery | RRI (0.670) | 23 (38.3) | 15 | 13 | 8 | 24 |
| Haitsma Mulier 2018 [14] | The Netherlands | ICU | 99 | 67.5 | 70.7 | Shock with MV | RRI (0.740) | 49 (49.5) | 26 | 6 | 23 | 44 |
| Darmon 2018 [15] | France | ICU | 351 | 62.1 | 63.2 | MV | RRI | 136 (38.7) | 68 | 69 | 68 | 146 |
|  |  |  |  |  |  |  | Semi-quantitative PDU (1.0) |  | 53 | 54 | 83 | 161 |
| Zhi 2019 [16] | China | ICU | 101 | 68.0 | 63.4 | Sepsis | RRI (0.692) | 21 (20.8) | 15 | 17 | 6 | 63 |
|  |  |  |  |  |  |  | Semi-quantitative PDU (1.0) |  | 17 | 7 | 4 | 73 |
| Zhi 2019 [17] | China | ICU | 63 | 69.0 | 66.7 | Cardiac surgery and sepsis | RRI (0.675) | 32 (50.8) | 19 | 8 | 13 | 23 |
| Zhi 2020 [18] | China | ICU | 148 | 67.0 | 58.1 | Cardiac surgery and sepsis | RRI (0.673) | 37 (25.0) | 29 | 37 | 8 | 74 |
|  |  |  |  |  |  |  | Semi-quantitative PDU (1.0) |  | 23 | 13 | 14 | 98 |
| Garnier 2020 [19] | France | ICU | 100 | 65.7 | 66.0 | Critically illness | RRI (0.685) | 50 (50.0) | 39 | 5 | 11 | 45 |
| Wiersema 2020 [20] | The Netherlands | ICU | 371 | 62.0 | 65.8 | Critically illness | RRI (0.740) | 123 (33.2) | 89 | 169 | 34 | 79 |
| Fu 2020 [21] | China | ICU | 107 | 70.5 | 66.4 | Sepsis | RRI (0.695) | 59 (55.1) | 45 | 25 | 14 | 23 |
| Zhi 2021 [22] | China | ICU | 83 | 69.0 | 66.3 | Cardiac surgery and sepsis | RRI (0.659) | 30 (36.1) | 22 | 15 | 8 | 38 |
|  |  |  |  |  |  |  | Semi-quantitative PDU (1.0) |  | 19 | 5 | 11 | 48 |
| Shankar 2021 [23] | USA | ICU | 50 | 44.0 | 80.0 | Liver transplant | RRI (0.690) | 25 (50.0) | 22 | 2 | 3 | 23 |

*AAAD: acute Stanford Type A aortic dissection; AKI: acute kidney injury; ICU: intensive care unit; MV: mechanical ventilation; PDU: power Doppler ultrasound; RRI: renal resistive index; TAVI: transcatheter aortic valve implantation
