# Supplementary material for: Performance of resistive index and semi-quantitative power doppler ultrasound score in predicting acute kidney injury: A meta-analysis of prospective studies
Source: PLoS One. 2022 Jun 28;17(6):e0270623. doi: 10.1371/journal.pone.0270623 (PMC9239473; doi:10.1371/journal.pone.0270623)
Supplement: S2 Table — (DOCX) [file pone.0270623.s006.docx]

S2 Table. The quality of methodological assessed using Quality Assessment of Diagnostic Accuracy Studies 2 scoring system

| Study | Risk of Bias | | | | Applicability Concerns | | |
| --- | --- | --- | --- | --- | --- | --- | --- |
|  | Patient selection | Index test | Reference standard | Flow and timing | Patient selection | Index test | Reference standard |
| Bossard 2011 [1] | Low risk | High risk | Low risk | Low risk | Low risk | Low risk | High risk |
| Darmon 2011 [2] | Low risk | Unclear | Low risk | Low risk | Low risk | Low risk | High risk |
| Schnell 2012 [3] | Low risk | High risk | Low risk | Low risk | Low risk | Low risk | Unclear |
| Guinot 2013 [4] | High risk | High risk | Low risk | Low risk | Low risk | Low risk | Low risk |
| Schnell 2014 [5] | Low risk | High risk | Low risk | Low risk | Low risk | Low risk | High risk |
| Sinning 2014 [6] | Low risk | Unclear | Low risk | Low risk | Low risk | Low risk | Low risk |
| Kararmaz 2015 [7] | Low risk | High risk | Low risk | Low risk | Low risk | Low risk | Low risk |
| Marty 2015 [8] | Low risk | High risk | Low risk | Low risk | Low risk | Low risk | Low risk |
| Marty 2016 [9] | Low risk | High risk | Low risk | Low risk | Low risk | Low risk | Low risk |
| Qin 2017 [10] | Low risk | High risk | Low risk | Low risk | Low risk | High risk | Low risk |
| Wybraniec 2017 [11] | Low risk | High risk | Low risk | Low risk | Low risk | Low risk | Low risk |
| Hertzberg 2017 [12] | Low risk | Low risk | Low risk | High risk | Low risk | Low risk | Low risk |
| Regolisti 2017 [13] | Low risk | High risk | Low risk | Low risk | Low risk | Low risk | Low risk |
| Haitsma Mulier 2018 [14] | Low risk | High risk | Low risk | Low risk | Low risk | Low risk | Unclear |
| Darmon 2018 [15] | Low risk | Unclear | Low risk | Low risk | Low risk | Low risk | High risk |
| Zhi 2019 [16] | Low risk | High risk | Low risk | Low risk | Low risk | Low risk | High risk |
| Zhi 2019 [17] | Low risk | High risk | Low risk | Low risk | Low risk | Low risk | High risk |
| Zhi 2020 [18] | Low risk | High risk | Low risk | Low risk | Low risk | Low risk | High risk |
| Garnier 2020 [19] | Low risk | Unclear | Low risk | Low risk | Low risk | Low risk | Low risk |
| Wiersema 2020 [20] | Low risk | Unclear | Low risk | Low risk | Low risk | Low risk | Low risk |
| Fu 2020 [21] | Low risk | High risk | Low risk | Low risk | Low risk | Low risk | High risk |
| Zhi 2021 [22] | Low risk | High risk | Low risk | Low risk | Low risk | Low risk | High risk |
| Shankar 2021 [23] | Low risk | Unclear | Low risk | Low risk | Low risk | Low risk | High risk |
