# Supplementary figures and images for: Performance of resistive index and semi-quantitative power doppler ultrasound score in predicting acute kidney injury: A meta-analysis of prospective studies
Source: PLoS One. 2022 Jun 28;17(6):e0270623. doi: 10.1371/journal.pone.0270623 (PMC9239473; doi:10.1371/journal.pone.0270623)

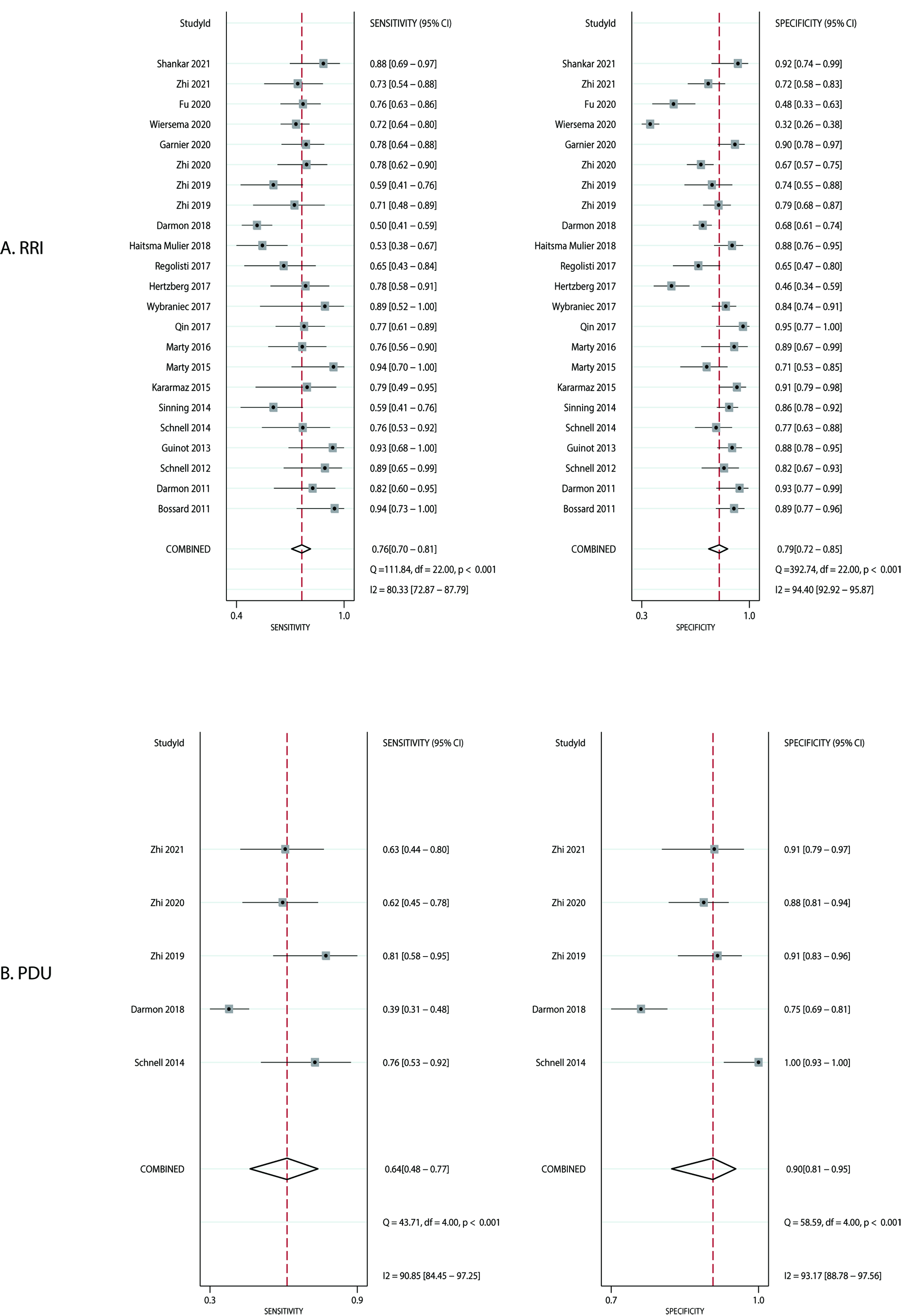

Supplement: S1 Fig — (TIF) [file pone.0270623.s007.tif]

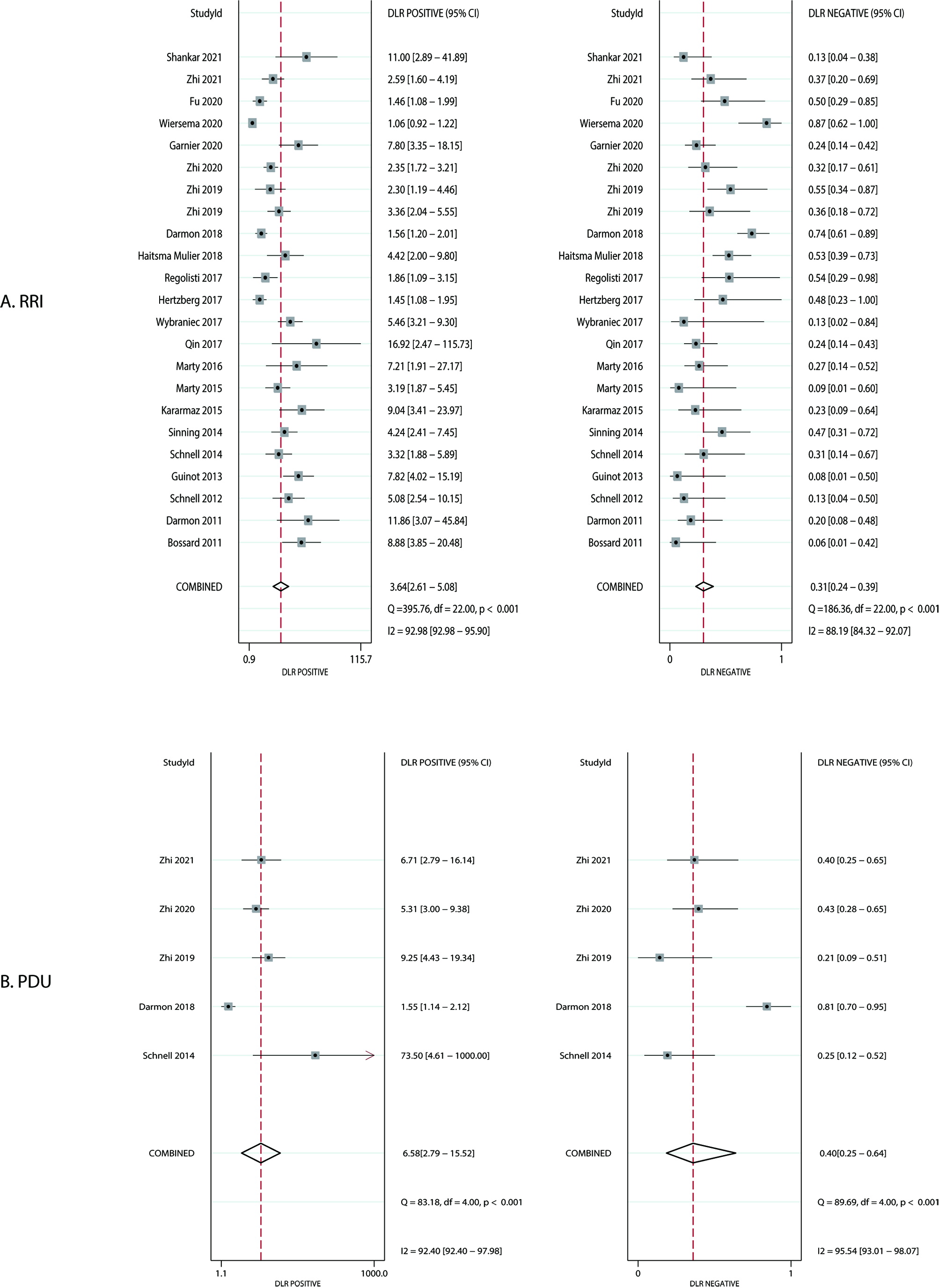

Supplement: S2 Fig — (TIF) [file pone.0270623.s008.tif]

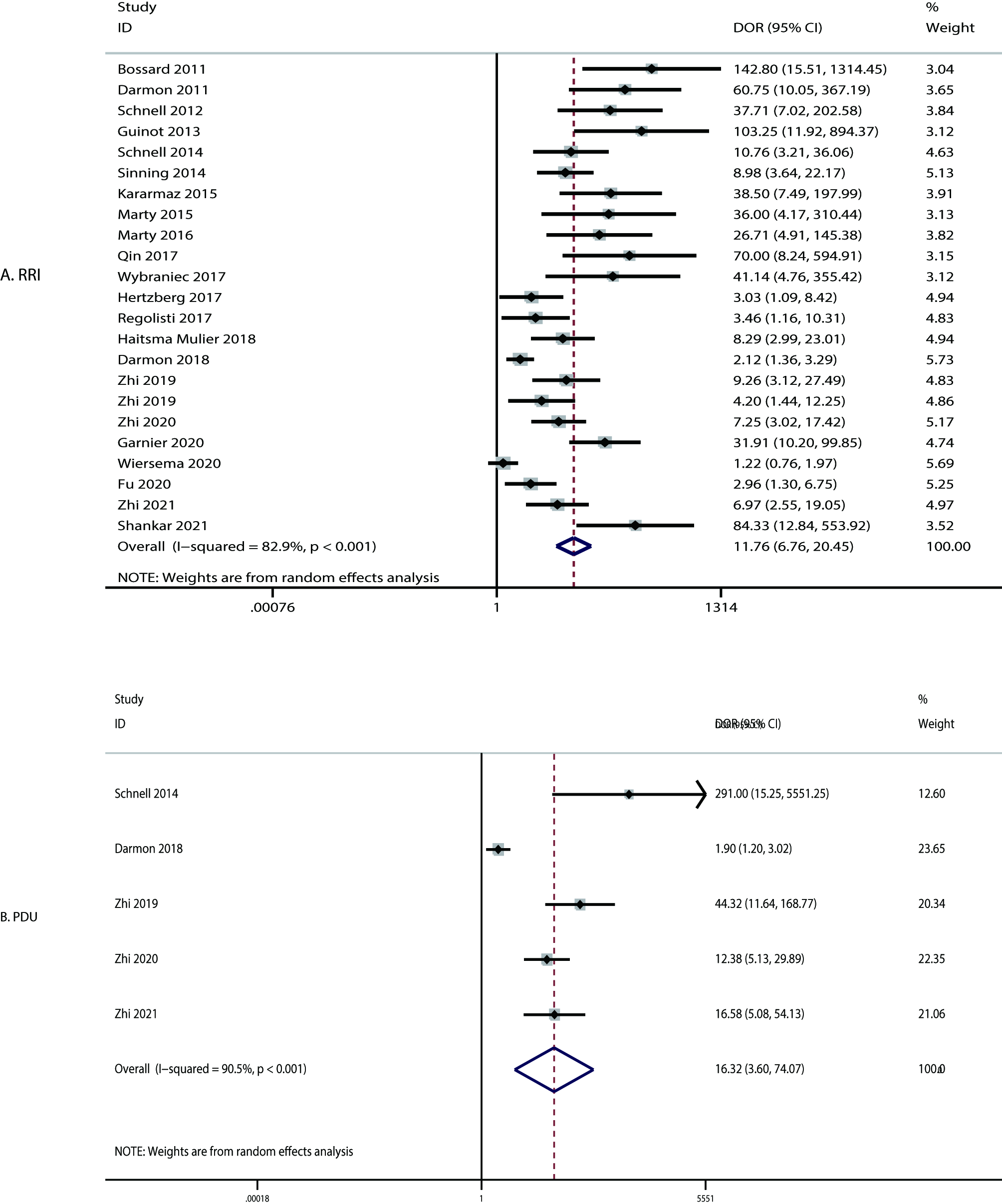

Supplement: S3 Fig — (TIF) [file pone.0270623.s009.tif]

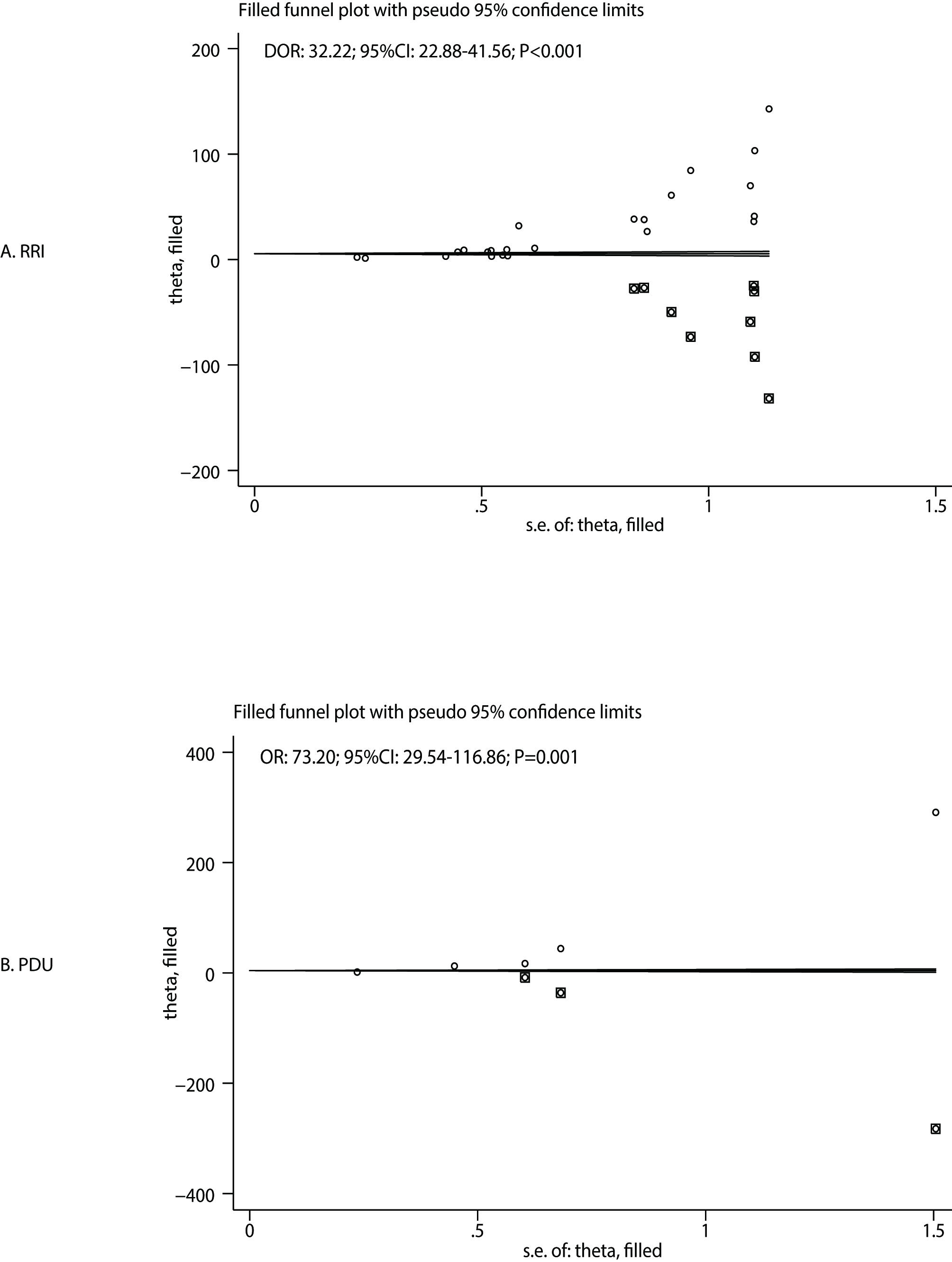

Supplement: S4 Fig — (TIF) [file pone.0270623.s010.tif]
